# Spatial dynamics of lithium battery recycling enterprises in China: implications for smart waste management and public health

**DOI:** 10.3389/fpubh.2025.1729413

**Published:** 2026-01-14

**Authors:** Feng Hu, Huijie Yang, Xiaolong Zhou, Shuang Zhao, Liping Qiu, Shaobin Wei, Jiahan Hu, Yufeng Chen, Hao Hu, Haiyan Zhou

**Affiliations:** 1 Institute of International Business & Economics Innovation and Governance, Shanghai University of International Business and Economics, Shanghai, China; 2 International Business School, Shanghai University of International Business and Economics, Shanghai, China; 3 School of Law, Shanghai University of International Business and Economics, Shanghai, China; 4 College of Business Administration, Ningbo University of Finance and Economics, Ningbo, China; 5 CEEC Economic and Trade Cooperation Institute, Ningbo University, Ningbo, China; 6 College of Engineering, University of Perpetual Help System Laguna, Biñan, Laguna, Philippines; 7 School of Economics and Management, Zhejiang Normal University, Jinhua, China; 8 School of Economics, Shanghai University, Shanghai, China; 9 Graduate School, Nueva Ecija University of Science and Technology, Cabanatuan, Philippines

**Keywords:** geographical detector, influencing factors, lithium battery recycling, public health risk, smart waste management, spatial pattern

## Abstract

The recycling of lithium batteries not only requires government guidance and support but also necessitates the deep involvement of enterprises to ensure sustainable production and promote circular economic development. Improper disposal and informal recycling of waste lithium batteries can release heavy metals and toxic electrolytes, posing potential threats to environmental safety and public health. Based on data related to lithium battery recycling enterprises in China, this paper utilizes spatial statistical methods and geographic detectors to analyze the spatial distribution patterns and influencing factors of these enterprises. The results indicated the following: (1) the overall spatial pattern of lithium battery recycling enterprises in China is characterized as having a “tiered” structure, with the eastern region consistently having the highest number of recycling enterprises, and a standard deviation ellipse shows the general temporal and spatial distribution trend in a south–north direction; (2) the spatial distribution of lithium battery recycling enterprises in China exhibits a significant positive spatial correlation, with a basically stable spatial pattern of cold and hot spots; Meanwhile, kernel density analysis indicates a gradual expansion of the spatial distribution, albeit with significant geographic polarization characteristics; (3) since 2013, lithium battery recycling enterprises have rapidly expanded, showing marked spatial differentiation within city clusters; and (4) the spatial distribution of these enterprises is significantly influenced by factors such as consumer spending levels and regional economic development, with all interaction factors showing a strong bi-factor enhancement effect. The findings provide guidance for developing smart waste-management systems to strengthen environmental governance and mitigate potential public health risks associated with lithium battery recycling.

## 1 Introduction

The promotion and use of new-energy vehicles are crucial for alleviating the current global climate warming and energy crises, and are key to China achieving its dual carbon goals (1). Lithium batteries are not only an important component of new-energy vehicles but are also widely used in devices such as mobile phones and computers, with the global market size and productivity of lithium batteries experiencing explosive growth (2). However, with the rapid development of the new-energy vehicle industry and the widespread application of lithium batteries, the reasonable recycling of retired lithium batteries has become an urgent new barrier to environmental protection (3). A 2025 report jointly released by Deloitte and CAS indicates that the volume of end-of-life power batteries will grow rapidly at a compound annual growth rate of 43% between 2021 and 2030.^1^ Waste lithium batteries are strategically valuable “waste,” and improper handling can not only cause ecological and environmental pollution but also hampers the green, sustainable supply and efficient reuse of critical mineral resources such as lithium and cobalt (4–7). These pollutants may cause ecological degradation and bioaccumulation, posing potential long-term threats to human health, particularly in densely populated urban regions. Therefore, examining where and how lithium battery recycling enterprises are spatially distributed is not only critical for industrial development but also for identifying potential public health exposure risks and guiding targeted interventions. Understanding the factors influencing this sector’s growth will help ensure its continued productivity and contribution to increased recycling and the circular economy, in keeping with UN Sustainable Development Goals, in particular SDG 7 regarding affordable and clean energy.

Informal recycling methods involve merely reconfiguring batteries with basic equipment to sell them to individuals for energy storage or modifying low-speed tricycles and motorcycles, or directly dismantling them to extract precious metals (8). Its dismantling process first releases fine particles containing heavy metals such as cobalt and nickel directly into the air, which can enter the human body through breathing, creating an initial exposure risk. Subsequently, more covert secondary pollution occurs. The heavy metals and electrolytes in the batteries enter water bodies and soil through rainwater leaching or untreated acidic wastewater, eventually accumulating in the human body through contaminated drinking water and agricultural products, leading to chronic poisoning and various long-term diseases. It is precisely these hazards that have prompted governments, enterprises, and research institutions to closely monitor how to design policies that guide and promote the formal recycling and reuse of waste power batteries (9).

Building on these concerns and policy drivers, recent developments in China further illustrate how formal recycling has become a national strategic priority. Given the substantial market potential of waste lithium batteries, major companies such as CATL and BYD have actively expanded into the recycling sector. The Chinese government has likewise intensified its policy support. In October 2024, it established China Resource Recycling Group Co., Ltd. to strengthen the circular economy and enhance mineral self-sufficiency. Complementary policy documents, such as the *Notice on Promoting the Coordinated and Stable Development of the Lithium-Ion Battery Industry Supply Chain*, encourage enterprise participation across the full battery life cycle. Despite this momentum, the industry remains in an early stage, with technological immaturity and limited large-scale recycling capacity. The Opinion of *the General Office of the State Council on Accelerating the Establishment of a Waste Recycling System* further highlights the need for a comprehensive recycling framework and strengthened traceability for power batteries. Currently, during the local implementation process in China, central-level strategies and policies have resulted in diverse subsidy standards, deposit pilot programs, and compliance requirements due to variations in regional economic development levels, industrial foundations, and regulatory resources (10, 11). This also underscores the necessity for regional analysis within this industry. Meanwhile, the environmental implications of improper recycling, particularly heavy-metal contamination, are underscored by studies on advanced leachate-treatment technologies such as EMBR (12, 13). Further driving this evolution, cutting-edge research is simultaneously focused on enhancing the sustainability of battery production itself, such as developing zero-waste synthesis pathways for key materials like LiFePO₄ (14). Together, these developments indicate that lithium-battery recycling in China is rapidly evolving into a government-led, industry-supported, and research-informed emerging sector.

An enterprise’s choice of location has always been a focal point of study within the field of economic geography. Classical location theory, profitability spatial boundary theory, and product lifecycle theory provide theoretical foundations and bases for research on enterprise location choices (15–17). Scholars primarily employ spatial statistical methods such as spatial autocorrelation models, spatial hot- and cold-spot analysis, standard deviational ellipse, and location entropy (18–20). While these methods effectively reveal clustering patterns, they often focus on manufacturing or service industries and remain limited in explaining why certain clusters form in environmentally sensitive sectors. Research has focused on urban, county, and provincial scales, studying entities like manufacturing and service-sector enterprises to explore the spatial distribution patterns and evolutionary rules of enterprises (21–23). Multi-scale spatial analyses offer meaningful conceptual guidance for this study, while a comprehensive analytical framework is essential for filling the current research gap in the spatial dynamics of the lithium battery recycling industry.

Furthermore, research on the determinants of firm location reveals an ongoing debate. Scholars generally believe that factors such as government support, labor costs, market size, economic foundation, and level of technological innovation have significant impacts on the spatial distribution of enterprises (24–26). However, there is relatively little research on the interaction between multiple influencing factors. For instance, Russia’s pharmaceutical enterprises are concentrated in the western region, heavily influenced by historical industrial centers (27). This indicates that when considering the factors of enterprise site selection, we cannot focus on the current time point and should also consider factors from a longer spatiotemporal perspective. Beijing’s high-tech enterprises are primarily distributed according to government planning and policy directives (28). China’s new energy vehicle enterprises exhibit a “T”-shaped distribution pattern, with policy, economic factors, and innovation serving as key determinants (29). As an emerging industry, these literatures provide support for us to select potential influencing factors. Additionally, China’s environmental enterprises exhibit hotspots concentrated in the east, while central and western regions hold development potential (30). Shipping service enterprises in the Yangtze River Delta cluster are hierarchically dispersed, with their locations influenced by the combined effects of transportation, information technology, and hinterland economies (31). According to existing literature, the factors that affect the location of enterprises vary depending on the industry, region, and historical environment. The research of scholars provides us with support, but due to the particularity of the industry and the characteristics of the times, it is necessary to study the factors determining the location of lithium battery recycling enterprises.

In summary, it can be seen that existing research has laid a solid foundation for corporate location choice. However, research on specific companies still shows deficiencies in terms of long-term evolution and multi-spatial scales, and the study of the interactions among influencing factors needs further enhancement. Specifically, research on lithium battery recycling has so far mainly focused on the importance and challenges of lithium battery recycling (32–35), and technological processes of such recycling (34–37), with studies on the spatial distribution of the related enterprises still being relatively unexplored. In view of this, this paper takes a geographical perspective and uses relevant data from lithium battery recycling enterprises in China as the research sample. Spatial statistical methods such as spatial autocorrelation and kernel density are used to study the spatial distribution and evolution characteristics of lithium battery recycling enterprises in China.

Based on this, the paper employs geographical detector analysis to identify influencing factors, which helps deepen current understanding of the distribution patterns of lithium battery recycling enterprises and provides theoretical guidance and practical basis for the development of China’s lithium battery recycling industry and the rapid development of related enterprises. The innovative points of this paper include enriching and expanding the research field of lithium battery recycling, offering references for the government to formulate and improve policies on the development of the lithium battery recycling industry, and promoting the development of the lithium battery recycling market. The findings of this paper will also aid in determining the optimal layout of future lithium battery recycling enterprises and promote the deepening of geographical theory research in provincial and urban agglomerations. In addition, the findings of this study contribute to the interdisciplinary understanding of how industrial geography and smart waste-management strategies intersect. By identifying the spatial hotspots of lithium battery recycling enterprises, the study provides a spatial basis for deploying smart monitoring, early-warning, and traceability systems to prevent environmental contamination and protect public health.

## 2 Research data and methods

### 2.1 Research data

The subjects of this study are lithium battery recycling enterprises registered within China. To start, the “Qixin Insight” platform’s batch search function was utilized to find enterprises related to lithium battery recycling and processing registered within China, using search terms such as “lithium-ion battery recycling and processing, lithium battery recycling, and waste lithium battery recycling.” The results identified 3,795 companies. Based on this, basic information on these enterprises was retrieved and supplemented from the company directory on the platform to form Table 1. It can be observed that limited liability companies dominate among China’s lithium battery recycling enterprises, with many having a registered capital of less than 10 million CNY. In terms of operational scopes, the top three industries are (1) wholesale and retail, (2) scientific research and technical services, and (3) manufacturing. It should be noted that the dataset reflects only legally registered enterprises, and no assumptions are made regarding their actual operational compliance or adherence to formal recycling standards. To further analyze the spatiotemporal evolution of China’s lithium battery recycling enterprises, directories of enterprises were formed according to the establishment and dissolution dates, covering the periods of 2003 and before, 2004–2008, 2009–2013, 2014–2018, and 2019–2023. Based on the timing of the promulgation and implementation of China’s policies on power battery recycling and the circular economy, the study period was divided into five stages. According to the enactment and subsequent revision of the Solid Waste Pollution Prevention and Control Law,^2^ together with the Several Opinions of the State Council on Accelerating the Development of the Circular Economy issued in 2005,^3^ the periods of 2003 and earlier and 2004–2008 were classified as the industry incubation stage. Guided by the Automobile Industry Adjustment and Revitalization Plan released in 2009,^4^ the period of 2009–2013 was identified as an expectation-driven growth stage, characterized by rising market anticipation of the industry. Based on the Development Plan for the Energy-Saving and New Energy Vehicle Industry (2012–2020),^5^ the period of 2014–2018 was defined as a stage in which the industrial system gradually took shape. Finally, in accordance with the Interim Provisions on Traceability Management of Recycling and Utilization of Power Batteries of New Energy Vehicles,^6^ the period of 2019–2023 was classified as the industrial system maturation stage.

**Table 1:** Basic information of lithium battery recycling enterprises in China.

Category		Quantity	Percentage
Type of enterprise	Joint-stock company	52	1.37%
	Limited liability company	3,623	95.47%
	Other	120	3.16%
Registered capital	<1 million	717	18.89%
	1 million–9.9999 million	1,626	42.85%
	10 million–50 million	966	25.45%
	>50 million	486	12.81%
Industry affiliation (top five)	Wholesale and Retail Industry	1,159	30.54%
	Science Research and Technology Services Industry	1,092	28.77%
	Manufacturing	986	25.98%
	Information transmission, software, and information technology services industry	199	5.24%
	Leasing and Commercial Services Industry	80	2.11%
Number of enterprises at various time periods	2003 and earlier	73	/
	2004–2008	171	/
	2009–2013	467	/
	2014–2018	2060	/
	2019–2023	3,726	/

Table 1 shows that China’s lithium battery recycling industry exhibits significant structural characteristics. In terms of legal form, limited liability companies dominate (95.47%), indicating that the industry is highly market-oriented and the responsibility boundaries are clear. The distribution of registered capital further reflects the stage of industry development: over 60% of enterprises have a registered capital of less than 10 million yuan, of which 42.85% are concentrated in the range of 1 million to 10 million yuan, showing an overall “light asset” start-up mode, reflecting the scale characteristics of the early stage of industry access. The industry classification presents a “three-legged” pattern: the wholesale and retail industry (30.54%), the scientific research and technology services industry (28.77%), and the manufacturing industry (25.98%) rank in the top three. This distribution is not only a statistical phenomenon, but also reveals the potential division of responsibilities in the industry chain, corresponding to core links such as the collection and circulation of battery recycling networks, technology research and development, and solution support, as well as dismantling and resource utilization. Overall, these characteristics depict an emerging industry landscape in the growth stage, with initial differentiation of market entities, but overall processing capabilities still need to be further improved.

### 2.2 Research methods

1. Spatial statistical methods. In this study, standard deviation ellipse, spatial autocorrelation, spatial hot and cold spots, and kernel density estimation are employed as spatial statistical methods. These tools are used to study the spatial distribution of centers, spatial clustering effects, and spatial association patterns of China’s lithium battery recycling enterprises from multiple perspectives. Detailed formulas can be found in the literature (38–42).
2. Geodetector. The factor detector module of Geodetector software was used to analyze the factors influencing the spatial distribution of lithium battery recycling enterprises in China. Furthermore, the factor interaction detector module was utilized to investigate the interactions of all factors affecting the spatial distribution of these companies at the chosen time point of 2023. Detailed calculation formulas can be found in the literature (43–45).

## 3 Analysis of the spatial pattern evolution characteristics of Chinese Lithium battery recycling enterprises

### 3.1 Spatial variation characteristics of the number of Lithium battery recycling enterprises

**Figure 1:**
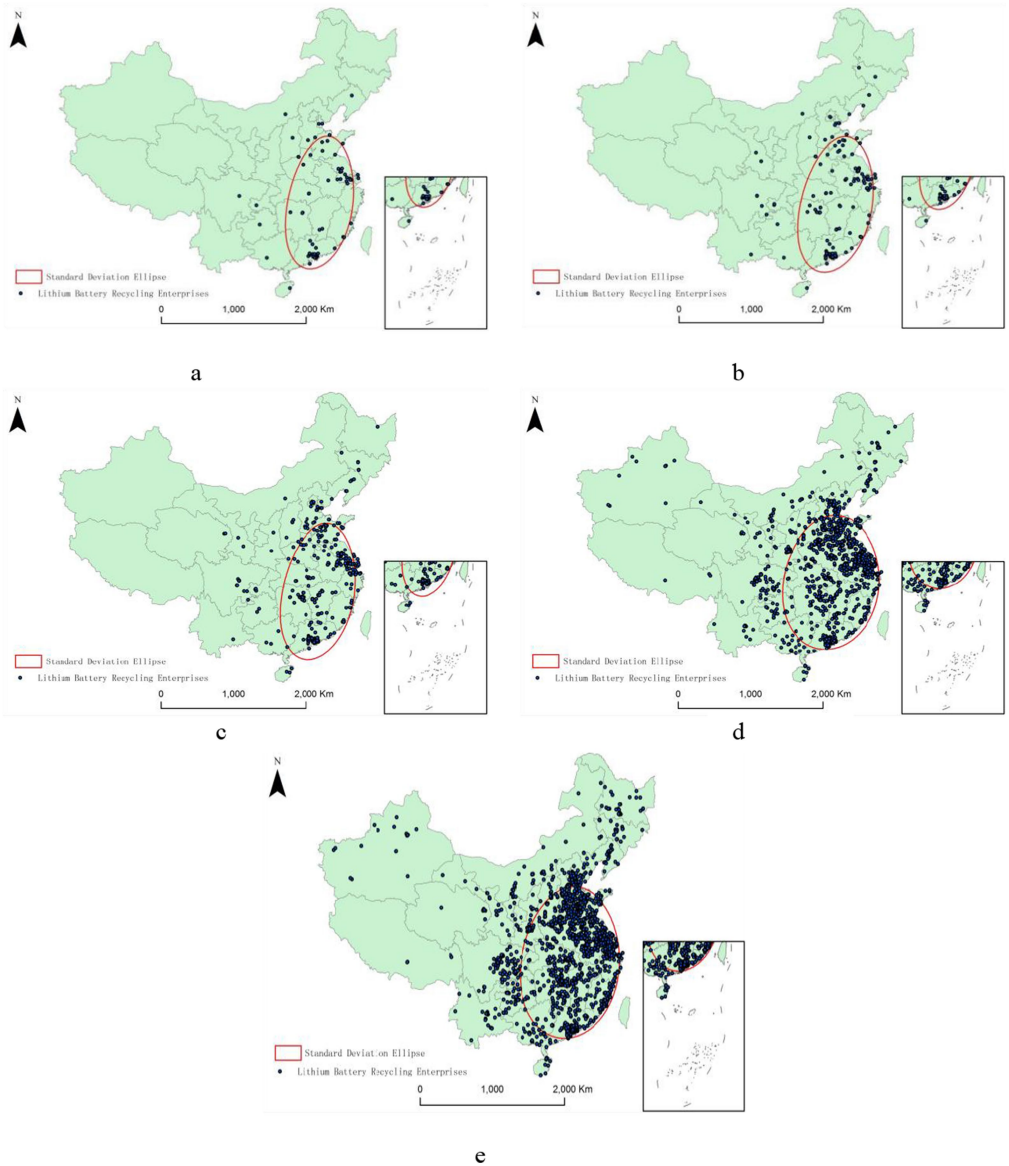
Spatial distribution of lithium battery recycling enterprises in China.

Figure 1 demonstrates that the spatial distribution of lithium battery recycling enterprises in China generally displays a “stepped” pattern. Specifically, from 2003 to 2023, enterprises in the eastern region consistently held the top position in terms of numbers, increasing from 52 to 1952. However, their percentage of the nation’s total decreased from 71.23 to 52.39%. Enterprises in the central region consistently ranked second, increasing from 16 in 2003 to 1,221 in 2023, with their national percentage rising from 21.92 to 32.77%. In the western region, the number of enterprises grew from 5 in 2003 to 553 in 2023, with their proportion increasing from 6.85 to 14.84%. Overall, the quantity growth was most substantial in the eastern region, considerably outpacing that in the central and western regions. In contrast, its trend in terms of their percentage increase is opposite to that of the central and western regions, indicating an ongoing diffusion of recycling capacity from coastal to inland areas. This shift helps balance regional waste management resources while also highlighting the need for regulatory oversight and environmental infrastructure development to extend simultaneously to emerging inland areas, thereby preventing the spread of potential risks. More specifically, at the provincial level, Guangdong and Jiangsu in the eastern region, along with Hunan and Anhui in the central region, and Sichuan and Shaanxi in the western region, consistently ranked at the top in terms of the number of enterprises. From a city perspective, Shenzhen, Changsha, Xiamen, Dongguan, Guangzhou, and Suzhou were continually in the top 10 for the number of recycling enterprises. Between 2019 and 2023, Hefei, Shijiazhuang, Fuzhou, and Xi’an entered the top 10 list. Given that these areas are hotspots for industrial activity and densely populated, it is reasonable to designate them as priority zones for environmental and health risk management. Consequently, the spatial distribution pattern of recycling enterprises not only highlights the opportunities for technological innovation to drive the industry’s prosperity but also underscores the necessity of integrating smart management and public health safeguards into the circular economy system in the future.

### 3.2 Characteristics of the standard deviation ellipse for Lithium battery recycling enterprises

The results of the standard deviation ellipse (Table 2) show that the distribution of lithium battery recycling enterprises in China generally exhibits a spatiotemporal distribution pattern oriented in a south–north direction. Starting from 21.50° before and including 2003, the orientation angle increased to 25.66° between 2019 and 2023. The axes of the ellipse have generally been growing, with the area of the ellipse expanding from 1,306,932.63 to 2,017,806.76 km^2^. This suggests that the spatial distribution pattern of Chinese lithium battery recycling enterprises is shifting from a northeast–southwest direction and becoming more dispersed. The spatial distribution of these enterprises is also becoming more uniform: the center of the ellipse has gradually moved northward from Jiujiang to Yichun in Jiangxi, then shifted westward to Huangshi in Hubei, and finally moved to Wuhan. This indicates that the spatial layout of the lithium battery recycling enterprises is developing towards regions with higher economic development levels.

**Table 2:** Standard deviation ellipse of spatial distribution for lithium battery recycling enterprises in China.

Related attributes	2003 and earlier	2004–2008	2009–2013	2014–2018	2019–2023
Azimuth/°	21.500384	24.237509	22.465323	24.545252	25.665095
Long axis standard distance/Km	8.462037	8.702387	8.665419	8.537268	8.679798
Short axis standard distance/Km	4.521205	4.906541	4.927157	6.733019	6.932564
Central location	Jiangxi Jiujiang	Jiangxi Jiujiang	Jiangxi Yichun	Hubei Huangshi	Hubei Wuhan
Elliptical area/Km^2^	1306932.63	1465555.67	1472161.17	1937091.16	2017806.76

### 3.3 Spatial correlation characteristics of Lithium battery recycling enterprises

From the results obtained using Moran’s I in Table 3, it can be observed that, except for the year 2003 and earlier, the spatial distribution of lithium battery recycling enterprises in China shows a significant positive spatial correlation. This means that provinces with a high number of lithium battery recycling enterprises are adjacent to each other, and those with fewer companies are also close to each other. From the trend of changes in Moran’s I, it can be seen that its value initially increases and then decreases, indicating that the spatial agglomeration of lithium battery recycling enterprises in China first intensifies and then diminishes. After the 2008–2012 period, the distribution of these enterprises began to gradually balance out. This shift from concentration to dispersion signifies an expansion of the geographic scope of industrial activities, necessitating the establishment of a nationwide regulatory network to ensure that development in emerging regions complies with environmental standards. Therefore, as the industry evolves, establishing a nationwide intelligent monitoring and traceability system based on emerging technologies such as IoT sensing and digital tracking is crucial. This will ensure that the spatial dispersion of recycling activities does not result in the geographical transfer of environmental and health risks.

**Table 3:** Spatial distribution of lithium battery recycling enterprises in China: Moran’s I value.

Year	2003 and earlier	2004–2008	2009–2013	2014–2018	2019–2023
Moran’s I	0.105454	0.156968	0.12924	0.04293	0.068108
*Z*-score	1.63776	2.557753	2.428415	1.661344	2.111337
*p*-value	0.101472	0.010535	0.015165	0.096644	0.034743

### 3.4 Spatial distribution hot- and cold-spot characteristics of Lithium battery recycling enterprises

**Figure 2:**
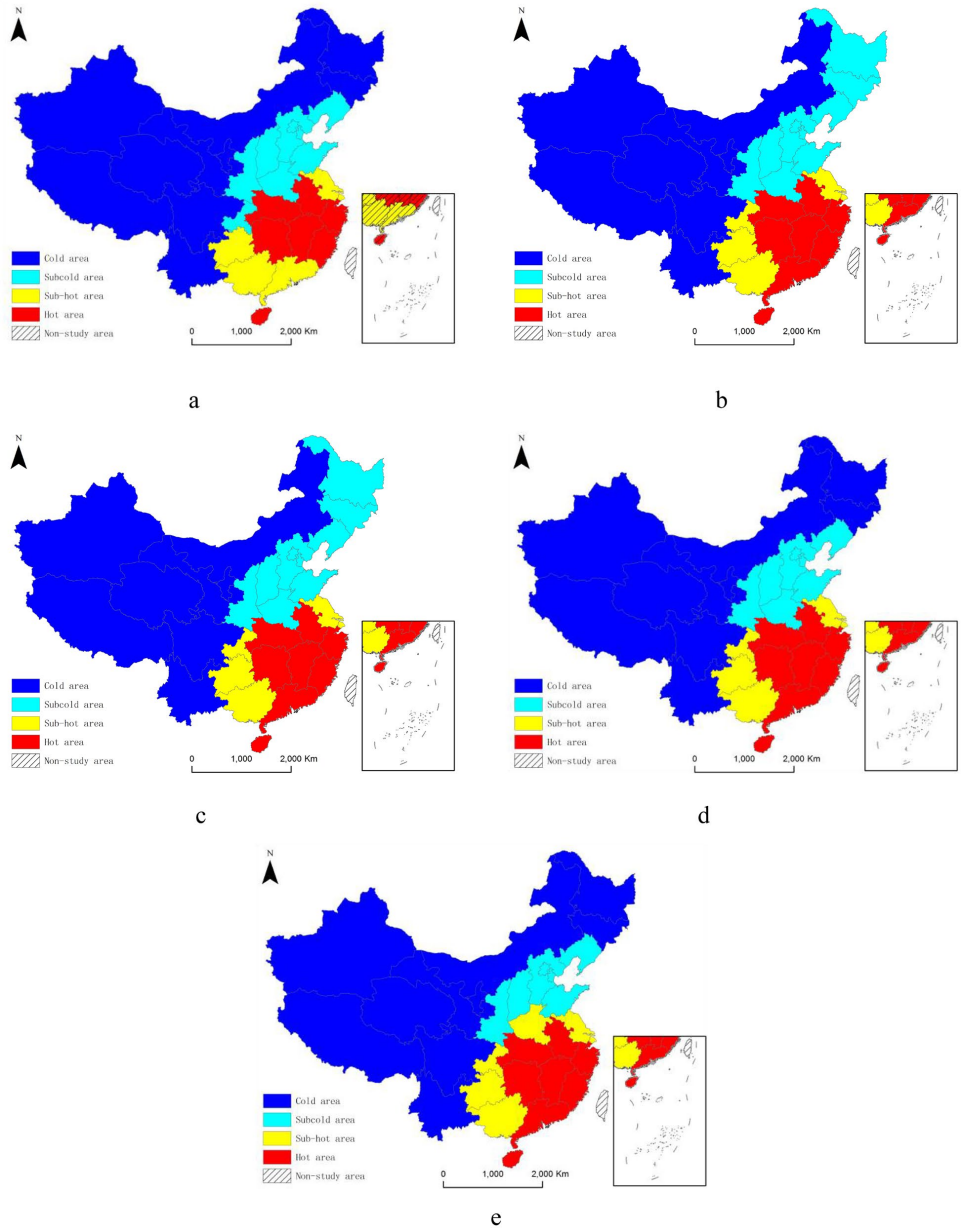
Spatial distribution of hot and cold spots of lithium battery recycling enterprises in China. **(a)** 2003 and earlier; **(b)** 2004–2008; **(c)** 2009–2013; **(d)** 2014–2018; **(e)** 2019–2023.

Further analysis of the spatial distribution of hot and cold spots among Chinese lithium battery recycling enterprises in Figure 2 reveals that before and including 2003, the hot-spot provinces were mainly Hubei, Anhui, Zhejiang, Hunan, Jiangxi, Fujian, and Hainan. Subsequently, Guangdong joined as a new hot-spot region, and the spatial distribution has remained relatively stable ever since. The sub-hot-spot provinces before and up to 2003 were Guizhou, Guangdong, Guangxi, and Fujian. After that period, aside from Guangdong becoming a hot spot, Chongqing and Henan were added as new sub-hot spots. The distribution of cold- and sub-cold-spot provinces has remained relatively stable, encompassing provinces mostly situated in the western and northeastern regions of China. These stable hotspot distribution patterns provide spatial guidance for governments to formulate nationwide public health safeguards, such as how to balance recycling activities across the country through smart-waste management and recycling technology.

### 3.5 Spatial distribution and kernel density analysis of Lithium battery recycling enterprises

Kernel density analysis using ArcGIS software reveals that the spatial distribution of lithium battery recycling enterprises in China has been expanding gradually. However, a significant geographic polarization feature is evident, characterized by expansion from a polarization point outward, eventually forming clustered structures and a zonal spatial pattern. The density of enterprise distribution has continuously increased, from a maximum of 0.8237 units/km^2^ in and before 2003 to a peak of 29.8991 units/km^2^ for the period from 2019 to 2023. Meanwhile, the polarization points have shifted from a dual-core to a single-core and then back to a dual-core configuration.

Based on the enterprise distribution density, enterprises are divided into five categories from low to high: low-density area, lower-density area, medium-density area, higher-density area, and high-density area.

**Figure 3:**
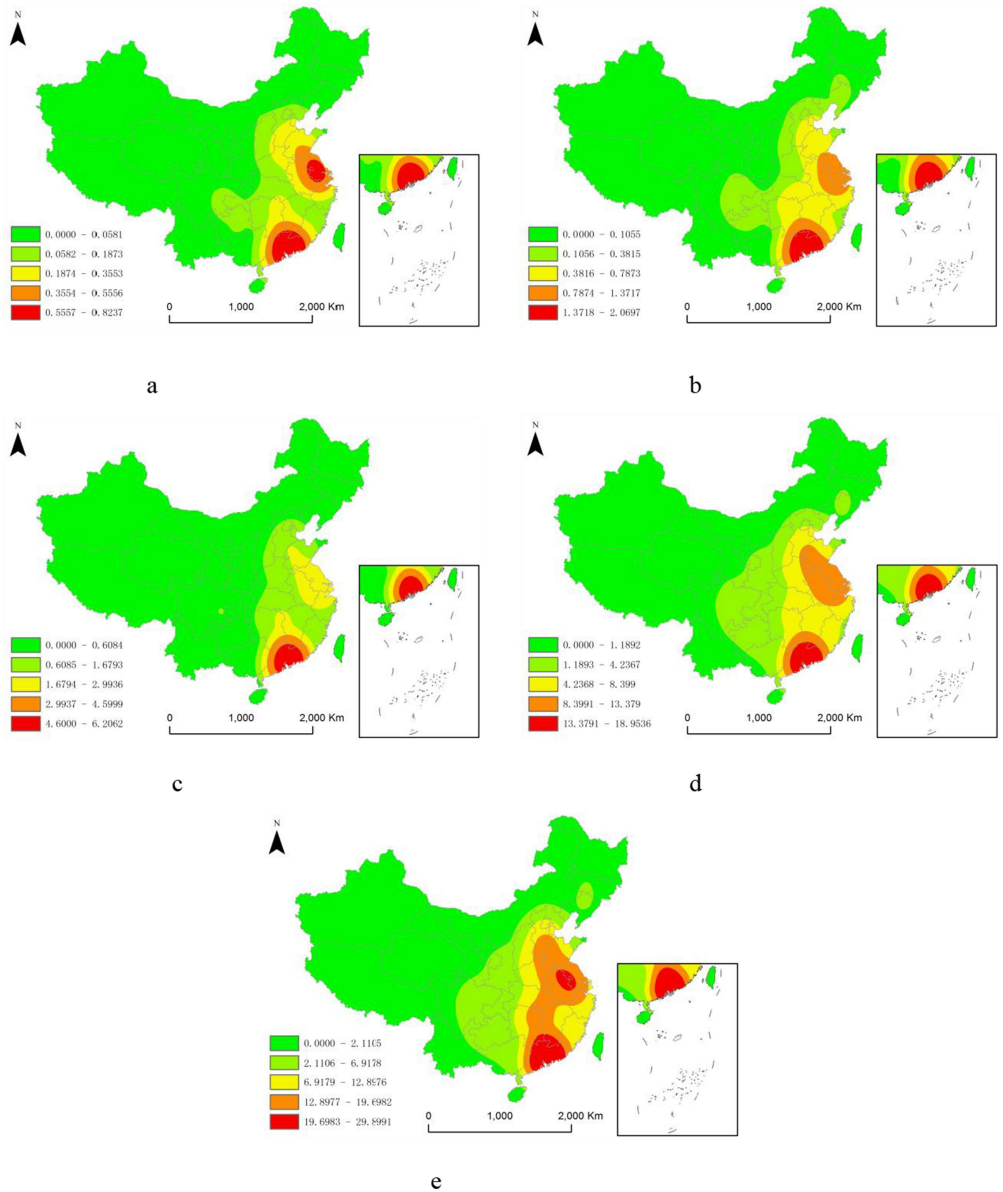
Spatial distribution of kernel density of lithium battery recycling enterprises in China. **(a)** 2003 and earlier; **(b)** 2004–2008; **(c)** 2009–2013; **(d)** 2014–2018; **(e)** 2019–2023.

As shown in Figure 3, in 2003, lithium battery recycling enterprises were mainly clustered in the eastern coastal regions of China, exhibiting a contiguous distribution trend of low density. The high-density areas were located in the Pearl River Delta and Yangtze River Delta regions, mainly involving the provinces of Guangdong and Jiangsu, with the Pearl River Delta being the largest cluster of lithium battery recycling enterprises. From 2004 to 2018, as the lithium battery recycling industry continued to develop, the Pearl River Delta remained the area with the highest density of lithium battery recycling enterprises. The distribution between this center and the sub-center, the Yangtze River Delta, evolved from a contiguous lower-density distribution to a medium-density distribution.

Starting in 2019, two high-density enterprise clusters of lithium battery recycling enterprises formed, one in the Pearl River Delta and the other in the Yangtze River Delta, with a contiguous higher-density distribution between them. Within the Pearl River Delta cluster, the high-density area has gradually extended northward to the southern parts of Hunan and Jiangxi provinces. In the Yangtze River Delta cluster, the high-density area has shifted from mainly Jiangsu Province to Anhui Province.

### 3.6 Spatial pattern evolution analysis of Lithium battery recycling enterprises in the five major coastal city clusters in Eastern China

Figures 1–3 show that the eastern coastal regions are the main distribution areas for China’s lithium battery recycling enterprises. Further calculations reveal that in each year, the five major city clusters of the eastern coast continue to be home to more than 50% of China’s lithium battery recycling enterprises, making these areas crucial core areas for the country’s economic and innovative development. Therefore, it is necessary to analyze the spatial pattern of lithium battery recycling enterprises from the perspective of these city clusters to achieve a more profound understanding of the spatial agglomeration drivers and effects.

**Figure 4:**
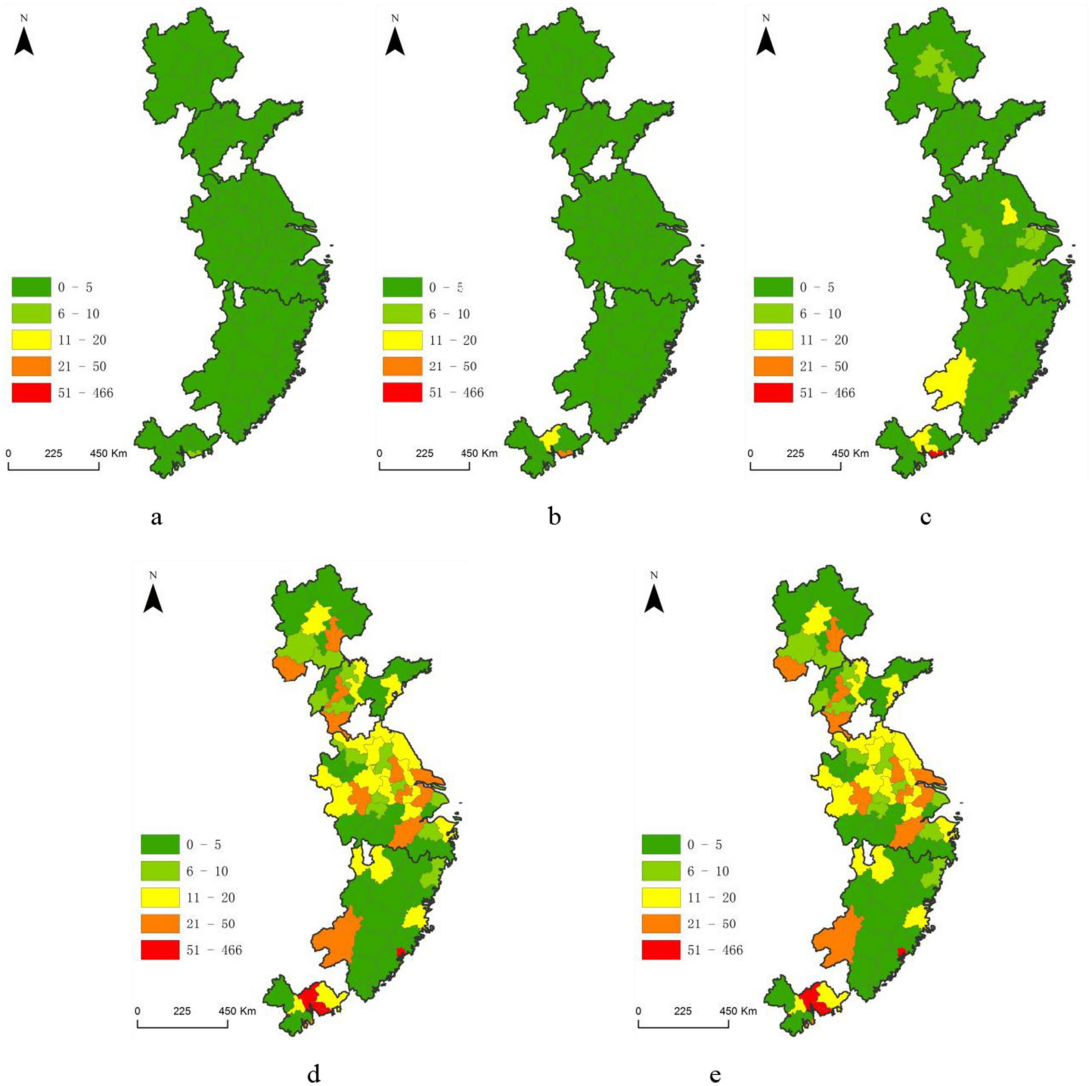
Spatial pattern of lithium battery recycling enterprises in the five major coastal city cluster in Eastern China. **(a)** 2003 and earlier; **(b)** 2004–2008; **(c)** 2009–2013; **(d)** 2014–2018; **(e)** 2019–2023.

Based on the number of companies, these areas are classified into five categories from smallest to largest: low-value areas, sub-low-value areas, medium-value areas, sub-high-value areas, and high-value areas. As shown in Figure 4, before 2008, only Shenzhen in the Pearl River Delta city cluster upgraded from a sub-low-value area to a sub-high-value area in terms of the number of lithium battery recycling enterprises, while Guangzhou became a medium-value area, and the remaining city clusters remained low-value areas. From 2009 to 2013, only Shenzhen in the Pearl River Delta city cluster became a high-value area, and the medium-value areas increased to now include Yangzhou in the Yangtze River Delta, Ganzhou on the west coast of the Strait, and Dongguan in the Pearl River Delta. Cities like Xiamen, Suzhou, Hangzhou, Tianjin, Wuxi, Hefei, and Beijing were classified as sub-low-value areas, while the Shandong Peninsula remained a low-value area.

From 2014 to 2018, Shenzhen, Guangzhou, Xiamen, and Dongguan became high-value areas for the distribution of lithium battery recycling enterprises, and the sub-high-value regions increased by 12 cities, including Hefei, Suzhou, and Yangzhou. From 2019 to 2023, four more cities became highly classified as high-value areas: Hefei, Shijiazhuang, Fuzhou, and Suzhou, and 10 more cities became sub-high-value areas, such as Nanjing and Xuzhou. All city clusters have a high-value area except the Shandong Peninsula. Overall, since 2013, lithium battery recycling enterprises have rapidly expanded, with significant leaps in the distribution tiers of cities within each city cluster. The development of lithium battery recycling enterprises in the eastern coastal city clusters has evolved from the Pearl River Delta northwards, showing significant spatial differentiation within each city cluster.

These coastal urban clusters feature a high concentration of enterprises, making them key areas for potential cumulative environmental pressures and management interventions. Of particular concern is the potential for heavy metal leaching into groundwater, as the cumulative effect of intensive recycling operations increases the likelihood of soil contamination and aquifer pollution (13). Since these city clusters predominantly occupy hydrologically vulnerable alluvial plains, critical sources of drinking and irrigation water, with any large-scale groundwater contamination should be regarded as priority areas for groundwater protection, where any potential pollution leakage could have far-reaching consequences. Thus, the identified agglomeration patterns delineate not only economic hotspots but also priority zones for groundwater protection, underscoring the urgent need for stringent spatial planning and environmental regulation in this emerging industry.

## 4 Analysis of the factors affecting the spatial distribution of Chinese Lithium battery recycling enterprises

### 4.1 Factors influencing the spatial distribution of Chinese Lithium battery recycling enterprises

Multiple factors have influenced the spatial distribution of lithium battery recycling enterprises in China. Following previous studies (26, 46–48), variables were selected across several dimensions for analysis in this paper. In the economic dimension, the total retail sales of consumer goods (X1) representing residents’ consumption level, GDP (X2) representing the level of economic development, and the total import and export trade (X3) representing the degree of regional openness were chosen. In the policy dimension, general public service expenditure (X4), representing government intervention, and the proportion of general environmental protection expenditure to GDP (X5), representing regional environmental awareness, were selected. In the innovation dimension, variables include the number of college students (X6) representing the level of human capital, the R&D expenses of large-scale industrial enterprises (X7) indicating the extent of research investment, and the number of patent applications (X8) representing innovation output. From the perspective of public health and waste management, understanding these factors is equally crucial, as economic prosperity, policy support, and innovation capacity collectively shape the spatial foundation of industrial activities, the very geographic framework within which smart management and risk mitigation measures are deployed.

These data were sourced from the China Statistical Yearbook and various provincial statistical yearbooks. The analysis was conducted at the end of different stages, specifically the years 2003, 2008, 2013, 2018, and 2023. Before performing Geodetector analysis, the related data were categorized into five levels using ArcGIS.

### 4.2 Analysis of influencing factors

Using the single-factor detection module of Geodetector, the analysis results (Table 4) indicate that the core influencing factors are the residents’ consumption level, economic development level, and the extent of scientific research investment, with other influencing factors considered secondary.

**Table 4:** Analysis of factors affecting the spatial distribution of lithium battery recycling enterprises in China.

Influencing dimensions	Variables	Specific indicators (abbreviated)	2003 and earlier	2004–2008	2009–2013	2014–2018	2019–2023
Regional economy	Residential consumption	Total retail sales of consumer goods (X1)	0.7281^***^	0.6465^***^	0.6390^***^	0.6187^***^	0.7614^***^
	Economic development	GDP(X2)	0.5716^**^	0.6634^***^	0.7088^***^	0.6379^***^	0.7327^***^
	Openness	Total Import and Export Trade Volume (X3)	0.4446	0.4425	0.4086	0.1986	0.5604^**^
Local policies	Government intervention	General public service expenditure (X4)		0.6526^***^	0.5715^***^	0.7208^***^	0.5742^**^
	Environmental awareness	The proportion of general environmental protection expenditure in GDP (X5)		0.3080^**^	0.4663^**^	0.4022^**^	0.3048^**^
Regional innovation	Human capital	Number of students in higher education institutions (X6)	0.3769	0.4975^**^	0.5957^***^	0.6468^***^	0.7037^***^
	Research investment	R&D expenses of large-scale industrial enterprises (X7)	0.7024^***^	0.6365^***^	0.6941^***^	0.6879^***^	0.7393^***^
	Innovative output	Number of Patent Applications (X8)	0.4059	0.4814^**^	0.5876^**^	0.6466^***^	0.6118^**^

“***” indicates significance at the 1% level, “**” indicates significance at the 5% level, and “*” indicates significance at the 10% level.

The influence of regional economies has been rising amidst fluctuations. The Q-values for resident consumption levels, economic development levels, and degree of openness show an upward trend, albeit with fluctuations. In particular, the degree of openness was significantly regressive in 2023. This suggests that the development of the lithium battery recycling industry has been greatly influenced by the regional residents’ consumption and economic development levels. Regions with higher economic activity and consumption levels also tend to generate larger volumes of used batteries. Concentrating recycling capacity in these areas helps reduce transportation-related emissions and potential environmental leakage, thereby improving both management efficiency and public health protection. In addition, upon reaching a certain stage, increased regional openness also began to significantly enhance the siting effect for lithium battery recycling enterprises.

The influence of regional policies has weakened amid fluctuations. The Q-values for government intervention and environmental awareness showed cyclical changes during the study period, generally displaying a downward trend. As the market potential of lithium battery recycling enterprises continues to increase, the number of enterprises also changes; hence, the impact of regional policies on the distribution of lithium battery recycling enterprises shows corresponding cyclical changes. It can be seen that the influence of regional innovation is slowly rising despite fluctuations. Regional innovation levels are crucial for competitiveness; the scientific and rational recycling of lithium batteries requires strong technological support, hence the Q-values for regional innovation are at higher levels. Moreover, regions with stronger innovation capacity are more capable of developing and adopting smart recycling technologies—such as AI-assisted sorting and IoT-enabled monitoring—which enhance operational safety and reduce human exposure to hazardous materials.

**Figure 5:**
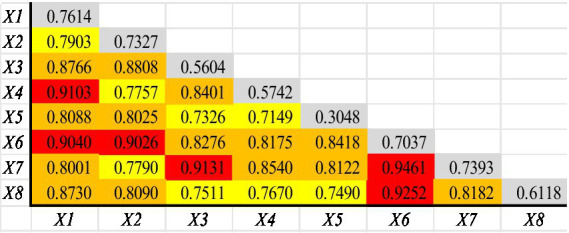
Results of the spatial distribution interaction factor detection for Chinese lithium battery recycling enterprises.

Using the interaction detection module of the geographical detector to analyze the years 2019–2023 (Figure 5), all interactive factors are found to have a significant bi-factor enhancement effect on the spatial distribution of lithium battery recycling enterprises in China. Among these factors, the strongest influence comes from the interaction between the degree of scientific research investment and the level of human capital, with a Q-value of 0.946, which is the highest level. Following this are the interactions between innovation output and human capital level, scientific research investment and degree of openness to the outside world, human capital level and consumer spending level, and human capital level and economic development level, as well as government intervention and consumer spending level, all of which have Q-values exceeding 0.9, categorized as the second highest level.

Other interactions, such as between the degree of openness to the outside world and consumer spending level, have Q-values less than 0.9 but all above 0.7, and are thus classified as the middle level. The effects produced by the interaction of these factors with other factors are greater than those produced by single factors alone, indicating that factors like consumer spending level need to be combined with other influencing factors such as economic development level to have a greater impact.

Regional innovation is a core interactive factor. The interaction between the extent of scientific research investment and the level of human capital has the strongest influence, exhibiting a dual-factor enhancement effect on the spatial distribution of lithium battery recycling enterprises in China. This indicates that provinces with higher levels of scientific research investment and human capital are more conducive to the development of lithium battery recycling enterprises. Additionally, the interaction of levels of human capital with levels of residents’ consumption and regional economic development are ranked second and third, respectively, suggesting that the innovation dimension plays a significant role in influencing regional economic aspects. It is evident that regional innovation significantly contributes to the regional economy and plays a crucial role in the site selection distribution of lithium battery recycling enterprises.

The influence of a region’s economy and regional policies is significantly enhanced when interacting with other influencing factors. For instance, after interacting with other factors, the Q-values of secondary influencing factors such as environmental protection awareness and degree of openness to foreign markets increased from 0.3048 to 0.715–0.842 and from 0.5604 to 0.733–0.913, respectively. This indicates that environmental protection awareness and degree of openness have a relatively limited impact on the development of lithium battery recycling enterprises in China. However, when these factors are linked with other influencing factors such as research and development investment, their importance and influence significantly increase.

Overall, the spatial distribution of Chinese lithium battery recycling enterprises is jointly shaped by regional economic, policy, and innovation factors. Beyond defining industrial geography, these interactions also imply differentiated capacities for smart waste management and health-risk mitigation. Regions with stronger innovation–policy–economy synergies are more capable of deploying digital monitoring, intelligent collection, and traceability systems that reduce environmental pollution and safeguard public health.

## 5 Conclusion, recommendations, and areas for future research

### 5.1 Conclusion

Using relevant data from Chinese lithium battery recycling enterprises, this study employs analytic methods such as standard deviational ellipse, spatial autocorrelation, and geographic detector software to analyze the spatial distribution pattern and influencing factors of lithium battery recycling enterprises in China. The results indicate the following:

1. Overall, the spatial distribution of lithium battery recycling enterprises in China presents a “stepped” pattern, with the number of enterprises in the eastern region consistently ranking first. Guangdong and Jiangsu in the east, Hunan and Anhui in the central region, and Sichuan and Shaanxi in the west remained at the top of the rankings throughout the study period. The standard deviational ellipse showed a northeast–southwest directional shift in the spatial distribution of these enterprises, indicating a dispersed pattern. Over time, however, the spatial distribution of lithium battery recycling enterprises in China is becoming more uniform. This spatial shift also has implications for public health, as more evenly distributed recycling operations can reduce localized environmental risks associated with lithium battery disposal and enhance the overall effectiveness of waste management policies.
2. The spatial distribution of lithium battery recycling enterprises in China shows a significant positive spatial correlation. The spatial clustering trends of these enterprises first increased and then decreased. After the 2008–2012 period, the distribution of enterprises began to balance more evenly. The spatial distribution pattern of hot and cold spots stabilized, with hot spots mainly concentrated in the provinces of Hubei, Anhui, Zhejiang, Hunan, Jiangxi, Fujian, and Hainan. Kernel density analysis indicated an expanding spatial distribution, but with significant geographic polarization characteristics, forming two high-density enterprise clusters in the Pearl River Delta and Yangtze River Delta. Between these clusters, there is a sub-high density continuous distribution.
3. The five major coastal city clusters in the east are the main distribution areas for lithium battery recycling enterprises in China. Within each city cluster, the distribution of lithium battery recycling enterprises showed significant increases in number, reflecting growing capacity for managing lithium battery waste. The development of these enterprises in the eastern coastal city clusters shows a spatial trend gradually extending northward from the Pearl River Delta, with significant spatial differentiation in the distribution of enterprises within each city cluster.
4. The spatial distribution of lithium battery recycling enterprises in China is significantly influenced by various factors such as the level of consumer spending and regional economic development. All interactive factors have a significant dual-factor enhancing effect on the spatial distribution of lithium battery recycling enterprises in China.

### 5.2 Insights and recommendations

1. Recycling used lithium batteries not only helps protect the environment but is also very beneficial for the repeated and efficient utilization of mineral resources such as lithium. With the increasing use of products equipped with lithium batteries, the recycling and disposal of used lithium batteries have become urgent issues that need to be addressed. As important participants in this sector, lithium battery recycling enterprises require the attention and support from national and local governments, who should introduce relevant policies and regulations to promote the industry’s development and improve industrial standards.
2. It is important to consider the influence of various factors, such as regional innovation, policies, and economics, on the development of lithium battery recycling enterprises. By enhancing the level of regional innovation and formulating comprehensive industry policies, a better innovation and business operation environment for lithium battery recycling enterprises can be provided, thereby promoting the clustered development of related enterprises.
3. The roles of the Yangtze River Delta and the Pearl River Delta, as growth poles for lithium battery recycling enterprises, should be fully leveraged. By improving related linkage mechanisms and attracting enterprises to set up in economically less developed areas, the spatial distribution of lithium battery recycling enterprises can be optimized, which in turn will drive the development of surrounding related enterprises. Strategic deployment of smart recycling infrastructure in these growth poles and adjacent regions can stimulate cluster development, improve resource recovery rates, and reduce localized environmental hazards that may affect public health.
4. Establishing an intelligent and transparent recycling network is crucial for minimizing environmental leakage and safeguarding public health. Priority should be given to deploying smart waste management technologies in hotspot regions such as the Pearl River Delta and Yangtze River Delta. These technologies include IoT-based monitoring systems for collection and dismantling sites, AI-driven sorting systems, and blockchain traceability platforms. Cross-provincial cooperation mechanisms should be leveraged to promote the adoption of these smart waste management technologies in lithium battery recycling industries across less developed regions.
5. To mitigate the environmental and health risks posed by the agglomeration of lithium battery recycling enterprises, the following targeted measures are recommended: At the technical level, promote enclosed intelligent crushing systems and advanced wastewater treatment technologies (such as EMBR) to control the emission of heavy metals and toxic substances from the source; at the spatial planning level, guide the construction of new facilities away from ecologically sensitive areas and residential areas; at the management level, establish an intelligent traceability system for the entire industry chain to implement environmental supervision throughout the entire process.

### 5.3 Theoretical contributions

1. This paper uses a geographical perspective to analyze the spatial pattern evolution characteristics of lithium battery recycling enterprises and study the influencing factors. It enriches and extends the research field of lithium battery recycling, providing references for the government to formulate and improve policies to boost the development of the lithium battery recycling industry and recycling market (33, 35, 36).
2. This study conducts an in-depth analysis of the spatial distribution characteristics of lithium battery recycling enterprises at the provincial and urban agglomeration scales, which not only facilitates identifying the optimal layout of future lithium battery recycling enterprises but also contributes to the further development of theoretical research on the geography of provinces and cities (23, 49).
3. Beyond contributing to the theoretical advancement of regional economic geography, this study bridges industrial spatial analysis with smart waste-management and public health perspectives, demonstrating how spatial data and technology-driven governance can jointly inform sustainable development policies.

### 5.4 Research limitations and future prospects

1. The reliance on enterprise count data from administrative registries, while revealing the formal sector’s geographical footprint, offers limited insight into operational reality. This data cannot distinguish between active processors, trading intermediaries, or “shell” registrations, nor does it account for the informal activities. To overcome this, future research must integrate multi-source data, combine operational metrics (e.g., tax records, verified capacity), environmental monitoring data (e.g., heavy metal concentrations in local soil/water), and regulatory enforcement records. Such integration would enable a transition from mapping “potential clusters” to directly assessing environmental and compliance performance.
2. The explanatory power of our model is constrained by the macro-level and proxy variables used, which may not capture the complex, localized drivers of enterprise location. Factors like specific municipal land-use policies, hyper-local market dynamics for black mass or recycled cobalt, and logistical nuances are absent. Addressing this gap requires a refined, multi-scale analytical approach. Future work should incorporate granular data on local planning ordinances, subsidies, and industrial symbiosis opportunities. Employing mixed methods, such as coupling spatial econometrics with in-depth case studies or executive surveys, can uncover the underlying decision-making mechanisms and policy interactions that our macro-analysis cannot reach.
3. Our spatial analysis identifies zones of recycling activity but cannot directly quantify environmental or public health risk, a limitation inherent to the distance between the enterprise network and actual impact. Future studies should bridge this gap by forging explicit spatial links. Research can quantitatively correlate the density and type of recycling enterprises with high-resolution environmental monitoring data and public health statistics. Applying point distribution analysis techniques could further refine the understanding of spatial clustering mechanisms at a finer scale. Furthermore, developing methodologies to map and model the informal sector’s material flows and practices is essential for a complete risk panorama. Finally, evaluating the effectiveness of smart technologies (e.g., IoT traceability, AI sorting) deployed in this industry would transform our spatial findings into a framework for evidence-based, targeted governance.

## Data Availability

The raw data supporting the conclusions of this article will be made available by the authors, without undue reservation.

## Footnotes

1 CAS and Deloitte China. Lithium-ion Battery Recycling: Market and Innovation Trends for a Green Future. Deloitte and CAS, 2025. https://www.deloitte.com/cn/en/Industries/energy/perspectives/lithium-ion-battery-recycling-report.html

2
https://www.mee.gov.cn/ywgz/fgbz/fl/202004/t20200430_777580.shtml

3
https://www.gov.cn/gongbao/content/2005/content_64318.htm

4
https://www.gov.cn/zwgk/2009-03/20/content_1264324.htm

5
https://www.gov.cn/zwgk/2012-07/09/content_2179032.htm

6
https://www.miit.gov.cn/jgsj/jns/wjfb/art/2020/art_b56b23d884934687aca9b346cafecbc4.html
