# Correction: Spatial dynamics of lithium battery recycling enterprises in China: implications for smart waste management and public health

**DOI:** 10.3389/fpubh.2026.1945660

**Published:** 2026-08-03

**Authors:** Frontiers Production Office

**Affiliations:** Frontiers Media SA, Lausanne, Switzerland

**Keywords:** geographical detector, influencing factors, lithium battery recycling, public health risk, smart waste management, spatial pattern

An incorrect affiliation was erroneously written for reviewer Banani Ray Chowdhury. The correct affiliation is “Techno Bengal Institute of Technology, India”. The affiliation has now been assigned.

The original version of this article has been updated.

